# Illness in breastfeeding infants relates to concentration of lactoferrin and secretory Immunoglobulin A in mother’s milk

**DOI:** 10.1093/emph/eov002

**Published:** 2015-01-20

**Authors:** Alicia A. Breakey, Katie Hinde, Claudia R. Valeggia, Allison Sinofsky, Peter T. Ellison

**Affiliations:** ^1^Department of Human Evolutionary Biology, Harvard University, Cambridge MA 02138, USA, ^2^Department of Anthropology, Yale University, 10 Sachem St., New Haven CT 06511, USA

**Keywords:** lactation, milk immunofactors, infant illness, lactoferrin, sIgA, life history, biology

## Abstract

**Background and objectives:** This study aims to better understand the relationship between immune compounds in human milk and infant health. We hypothesized that the concentration of immune compounds in milk would relate to infant illness symptoms according to two possible theoretical paradigms. In the ‘protective’ paradigm, high concentrations of immune compounds prevent infant illness. The converse, the ‘responsive’ framework, posits that concentrations of immune compounds are elevated in response to infection.

**Methodology:** Milk samples (*n* = 110) and illness data were collected among the Toba of Argentina from 30 mother–infant dyads. Samples were assayed for two immune proteins, lactoferrin and secretory immunoglobulin A (sIgA). Generalized estimating equations were used to assess the relationship between immune composition of milk and symptoms of illness in infants.

**Results:** Lactoferrin was positively associated with symptoms of illness in infants (odds ratios >1), both in the month preceding the sample collection and the subsequent month. sIgA was negatively associated with symptoms (odds ratios <1) in the preceding and subsequent months, an association which was particularly strong for gastrointestinal symptoms.

**Conclusions and implications:** The two compounds investigated in our study had opposite relationships with symptoms of illness; the positive relationship between lactoferrin and illness lends support to our ‘responsive’ paradigm, and the negative relationship between sIgA and symptoms of illness was consistent with our ‘protective’ framework. That elevated lactoferrin is restricted to periods of illness suggests that there may be a cost to mother or infant associated with persistently elevated lactoferrin that is not incurred with elevated sIgA.

## INTRODUCTION

Breast milk composition is a critical and underexplored area of human biology. It is now widely accepted that, compared with formula feeding, breastfeeding provides several health and developmental benefits to infants in a variety of environments. These benefits include lower rates of infectious illness (reviewed in [1]), especially with regard to infant respiratory and gastrointestinal illness. In affluent developed nations, breastfeeding is associated with a reduced incidence and shorter duration of lower respiratory infections [2]. The protective role of breastfeeding is even more evident in developing countries, where infectious disease mortality is a greater threat to infants. For instance, one study of infants in a slum in Bangladesh found that infants who were not exclusively breastfed experienced a higher risk of death by respiratory infection and diarrheal illness [3]. The latter finding was reinforced by a recent meta-analysis of infant deaths attributable to diarrheal illness in the developing world which found a much higher relative risk of death to infants who were not exclusively breastfed during the first 6 months of life and at least partially breastfed until 2 years of age [4].

In addition to the clear public health implications of informing breastfeeding decisions, understanding the cause and consequence of the natural variation in milk composition that occurs between individuals, populations and species is an area of increasing interest to evolutionary biologists. By considering the complexities of variability in human milk composition, we ultimately seek to gain insight into life history tradeoffs of the mother and infant. Through the lens of life history theory we can evaluate tradeoffs in the allocation of resources among growth, reproduction and somatic maintenance [5]. Lactation is an interesting and complicated life stage from a life history perspective because the biologies of mother and infant interface and overlap, and understanding milk composition requires a consideration of the tradeoffs and investment decisions experienced by both individuals. For instance, if immune compounds in milk are elevated, it could be reflective of investment by the mother in her own somatic maintenance (especially if the compound is passively transported into milk), investment in reproductive effort (if it is expected to help the child survive to reproductive age) and perhaps even investment in the growth of her infant (if the compound helps the infant avoid an illness that might divert resources preferentially to maintenance).

Variation in macro- and micronutrient composition in human milk has been studied for decades [6, 7] but recent research has revealed the presence of many bioactive molecules and other constituents of milk whose functions are just beginning to be investigated [8, 9]. These compounds—including immune molecules, hormones, oligosaccharides and bacteria—present as good candidates for the regulation of the complicated immune modulation induced by milk. In addition to the recognition of and response to antigens by milk immune compounds, milk contains lymphocytes, cytokines, nucleotides and other compounds that promote priming of the naïve neonatal immune system [10].

Integrating the study of natural variation in milk and associated infant health outcomes brings breast milk research to the intersection of the fields of evolutionary biology and public health. The focus of the current investigation was to elucidate the relationships between concentrations of two immune molecules found in human milk—lactoferrin and secretory immunoglobulin A (sIgA)—and the development of symptoms of gastrointestinal and respiratory illness in the infants ingesting the milk.

Maternal–origin lactoferrin is produced in mammary epithelial cells [11] and secreted into milk. Once ingested by the infant, lactoferrin provides immune protection during early life while the infant’s immune system develops immunological competence. Lactoferrin is a multifunctional innate immune protein found at mucosal surfaces. It is capable of restricting the proliferation of infectious organisms in a variety of ways. Perhaps the best-known role of lactoferrin is to bind iron in the gastrointestinal tract, which sequesters it from pathogenic bacteria that need iron to proliferate [12]. Lactoferrin also contains a highly basic N-terminal domain called lactoferricin, which is capable of lysing bacterial cell membranes (reviewed by [13]). There are also many effects of lactoferrin on the action of other immune cells, including increasing motility of granulocytes, regulation of lymphocyte maturation, upregulating production of natural killer cells and influencing production of both pro- and anti-inflammatory cytokines [14]. Finally, lactoferrin can bind to host epithelial cells via the lactoferrin receptor and increase the resistance of those cells to intracellular invasion by pathogens by interfering with pathogen adhesion mechanisms [15]. While the role of lactoferrin is usually presented as antibacterial, lactoferrin is also capable of interfering with viruses, fungi and protozoa [16].

sIgA is another major source of immune protection transferred to the breastfed infant. Maternal B-cells are ultimately responsible for the production of IgA. These cells are primed to bind to specific antigens in the mother’s gastrointestinal and respiratory tracts and travel to mammary tissue via the lymphatic system, in a process known as ‘homing’ [17]. The protective action of sIgA in the infant gut occurs via many processes, including intracellular neutralization and excretion of viral particles; immune exclusion, whereby sIgA prevents the binding of a pathogen to a mucosal surface; agglutination of bacteria and viruses; and interference with bacterial motility [18].

Diarrheal disease, a leading cause of infant illness and death worldwide [19], is reduced among breastfed infants. A handful of studies have investigated the protective role of milk immune factors against infant diarrheal illness *in vivo*. Walterspiel *et al.* [20] reported that, among infants infected with *Giardia lamblia*, those infants who received higher concentrations of anti-*Giardia* specific sIgA from milk experienced fewer episodes of diarrhea, though there was no difference in the total concentration of sIgA between the milk of infants who were infected and those who were not. A similar finding was reported by Ruiz-Palacios *et al.* [21]: breastfed infants who developed diarrhea specifically as the result of infection by *Campylobacter jejuni* were found to lack sIgA molecules specific to *C. jejuni* antigens, although there was no difference in total (non-specific) sIgA in the milk between breastfed infants who developed *C. jejuni* diarrhea and those who did not. Addition of human recombinant lactoferrin and lysozyme to an oral rehydration solution was found to shorten the duration of illness among infants with diarrhea and dehydration in a randomized controlled trial [22]. Similarly, a study of formula-fed infants found that supplementation with bovine lactoferrin was associated with lower risk of illness, particularly wheeze and lower respiratory tract infections in the first year of life [23]. Hassiotou *et al.* [24] published a study of milk composition across lactation in urban Australian women that found that lactoferrin concentration remains unchanged in the event of maternal or infant illness, but sIgA slightly rises. With the exception of this single study, infant outcomes in relation to natural variation in milk lactoferrin concentration have not been previously published.

This study was designed to address some of the deficits in our understanding of individual variation in milk bioactive compounds and related infant outcomes. Here we test the hypothesis that natural variation in the concentration of the immune compounds lactoferrin and sIgA in human milk relates to the development of symptoms of illness in infants in an environment with relatively high exposure to pathogens, including gastrointestinal symptoms like diarrhea and vomiting, and respiratory symptoms like cough and mucus. We propose two theoretical frameworks to understand the relationship between immune compounds in milk and symptoms of infection. The *protective* paradigm posits that an initial elevation of immune compounds in milk will serve to fight infection, resulting in fewer symptoms of illness experienced by the infant. In this paradigm, symptoms of illness and concentration of immune compounds will have a negative relationship. Between mothers, higher levels of a preventive compound could be thought of as a biomarker of a healthy infant, as its elevated concentration in the milk is more likely to predict an infant with no symptoms. In contrast, the *responsive* paradigm posits that infection in the infant will be detected by the mother (through increased environmental exposure to the pathogen, or perhaps via immunological changes in the infant’s saliva detected by breast tissue), who will increase the transfer or production of immune compounds in the milk to the infant. In this framework, symptoms of illness and concentration of immune compounds will be positively related. Higher levels of a responsive compound may be thought of as a biomarker of an ill infant, as its elevated concentration in the milk would be more likely to predict an infant experiencing symptoms of illness.

We present these paradigms as logical frameworks rather than strictly temporal explanations, because the timeframe of milk sampling with respect to individual bouts of illness may obscure the temporal dimension of the relationship. Logically, then, a preventive compound is one that is upstream of infant health status, and a responsive compound is downstream. It is possible, and even likely, that the same compound may serve both a protective and a responsive role, especially when considered over an extended period of time. Ultimately, the effectiveness of elevating any individual compound in either a protective or responsive capacity will determine where along the protective–responsive continuum it is primarily categorized.

## METHODOLOGY

### Study population

We conducted our study among the indigenous Toba people of Namqom, Formosa Province in northeastern Argentina. Namqom is a village of ∼3500 Toba located 11 km northwest of the city of Formosa. The Toba, who have historically been hunter-gatherers, are experiencing dramatic changes in their lifestyle [25]. During the last century, disruptions to their traditional livelihood and ecological deterioration of the habitat have forced massive migrations to urban centers. While rural communities still use the forest as a source of food and shelter, families like the ones in Namqom live in poor peri-urban *barrios*. This population has free access to health services, mainly provided by the local health center and the city’s hospitals. The provincial government offers pre- and postnatal care programs.

We conducted this study in an environment with greater exposure to a variety of pathogens than generally found in the US or other regions of the developed world, reasoning that greater exposure to pathogens would highlight the relationships being tested. None of the homes in the community have indoor toilets, and there is standing water in ditches close to most homes containing sewage runoff. About half of homes have dirt floors, and infants are routinely placed on uncovered soil, both inside and outside the home. Drinking water is available through shared taps. Most homes do not have refrigerators, and much of the cooking is done over outdoor fires. These environmental conditions are known to increase risk of exposure to pathogens, and appreciably elevate the exposure risk above the typical environmental conditions found in the West.

Toba women typically breastfeed their children on demand for 2 or 3 years or until the next pregnancy is noticeable [26]. Co-sleeping also allows for on-demand nighttime nursing. Semisolid and solid supplements are usually introduced around 6 months of age [27]. Exclusive bottle-feeding is uncommon, though supplementing breastfeeding with cow’s milk is fairly common. For a more detailed report of the demographic profile of this population see [26]. This population has been studied extensively since 1997 as part of the Chaco Area Reproductive Ecology (C.A.R.E.) Program under the direction of Dr. Claudia Valeggia. In addition to providing the appropriate pathogenic environment in which to test our hypotheses, we chose to study the Toba because of the quality of the existing infrastructure and resources available to researchers at the field site in Namqom. The current study participants were recruited from the participants of Dr. Valeggia’s ongoing study of infant health and growth.

### Study dyads

Thirty breastfeeding infants and their mothers were recruited for this study. Study protocol was approved by the University of Pennsylvania Institutional Review Board (#811200), and subjects provided verbal consent at enrollment and each study visit. All infants recruited into the study had been born in the local hospital. Each mother–infant pair was visited approximately once a month for 4–5 months, until they weaned their infant, or indicated they wished to cease participation (of 30 participating dyads: four women participated in five visits; 18 women had four visits; four women had three visits; two women had two visits; and two women had one visit). Data collection took place over a 6-month period in 2012 and 2013.

Nine women (30%) were primiparous and 21 (70%) were multiparous (mean = 3.2 children, SD = 2.45, range 1–10). At recruitment, mothers ranged in age from 15 to 37 years (mean maternal age = 24.4 years, SD = 6.4 years). Infant age at the first study visit ranged from 35 to 448 days (mean infant age = 244 days, SD = 126 days). Of the recruited infants, 18 (60%) were female and 12 (40%) were male.

### Milk collection procedure

At each study visit, a hand-expressed mid-feed milk sample was collected using a test-weigh protocol. Mid-feed sampling produces a milk sample representative of a full mammary evacuation and only results in a minimal loss of milk to the infant, making it a more ethical method in a nutritionally stressed population [28]. Hand expression is preferable to using a breast pump due to logistical constraints in the field (such as keeping pump parts sterile), and was more culturally appropriate for our population, who do not typically use pumps. When the interviewer arrived, the mother was asked to nurse the infant from the breast opposite her dominant hand. Then nursing was restricted from that breast for the subsequent 2 h (the infant was free to feed from the other breast during those 2 h). At the end of the 2-h milk collection period, the infant nursed for 2 min from the study breast, and then a sample of 10 ml of breast milk was expressed into a collection tube and immediately mixed by gentle inversion and aliquoted into 1 ml screw-top storage vials. Samples were transported to the field station on ice and frozen within 2 h of collection. Samples were stored frozen until they shipped to the US on dry ice, where they were stored at −20°C.

### Interview data

During the 2-h milk collection period, an interview was conducted. Infant health at the time of the interview and over the month prior to the interview was assessed, with questions targeted at symptoms of gastrointestinal illness (diarrhea and vomiting) and respiratory illness (cough, cold, mucus). The interview also included questions about household demographics and supplementary feeding of the infant.

### Milk composition analyses

Laboratory analyses were conducted in the Harvard University Reproductive Ecology Laboratory. Lactoferrin in milk was assayed using an ELISA kit from ALPCO (#41-LACHU-E01). Following kit instructions, whole milk samples were diluted 1:50 000 with diluent. Samples that ran out of range were rerun at 1:100 000. All samples from an individual woman were run in the same plate to minimize the effect of interassay variation. Intraassay variability was 2.2%. The kit manufacturers report an interassay variability of <10%. No controls were provided for use with this kit, but standard curve values were consistent with values published on the kit insert. sIgA in milk was assayed using an ELISA kit from Salimetrics (#1-1602) intended for use with saliva. The kit protocol was followed exactly, with the exception that milk samples were diluted 1:5 with diluent. No samples ran out of range. All samples from an individual woman were run in the same plate to minimize the effect of interassay variation. Interassay variability was 11.7% and intraassay variability was 4.8%. Lactoferrin and sIgA values were log-transformed before analysis to normalize the distribution of values. One sample with extreme outlying values in both lactoferrin and sIgA was excluded from analyses for suspected contamination.

### Statistical analyses

Statistical analyses were conducted using SPSS 21. Generalized estimating equations (GEE) were performed with a binomial distribution, logit link function and exchangeable correlation structure. GEEs were chosen because they correct for the non-independence associated with repeat measures and allow for a binary outcome variable. The following variables were included as explanatory variables in the preliminary GEE models: log-transformed concentrations of lactoferrin and sIgA, change in concentrations of both compounds from the previous month expressed as a percentage of the previous month’s concentration, infant age (continuous), infant sex, maternal parity (continuous) and floor type (dirt or cement) of the infant’s home. Infant age, maternal parity and floor type were never significantly related to infant illness, and were removed. This resulted in an improvement in the QICc values of our models and these variables were permanently excluded. The outcome variables were symptoms of infant illness (binary) at different times of interest.

Analyses were conducted for three time periods: symptoms in the month preceding the study visit, symptoms at the time of the study visit and symptoms in the month following the study visit. (The data about future symptoms of illness were obtained at the following month’s interview.) Symptoms were further separated into gastrointestinal, respiratory and total. This resulted in a total of nine analyses. Significance was set at *P* < 0.05. Results presented (Table 3) have been corrected for multiple models using the Benjamini–Hochberg false discovery method. Because some of the explanatory variables (monthly changes in immune compounds) relied on having information from previous or subsequent visits, the 110 study visits resulted in *n* = 79 full data points for past and current symptoms, and *n* = 52 data points for subsequent month’s symptoms.

Plots of Pearson residuals versus predicted responses were examined for outliers and heterogeneity. Four of the nine models had one outlier each. Outlying points (those with a residual greater than three standard deviations) were removed and analyses rerun. The direction and absolute significance of results at *P* = 0.05 did not change when outliers were removed; however, the odds ratios were made more extreme. Because the outliers may represent meaningful variation in the population and do not change the interpretation of results, results presented include all data points. Plots did not reveal a clear heterogeneity of data and histograms of residuals were relatively normally distributed.

## RESULTS

All infants enrolled in the study experienced illness during the study period; *N* = 30/30 (100%) infants were reported as having illness at any of their home visits. Of infants with more than one study visit, 27/28 (96%) experienced repeated bouts of illness. The incidence of symptoms varied by subtype (respiratory and gastrointestinal) and period of inquiry (symptoms concurrent with study visit or in previous month) (Table 1). Both lactoferrin and sIgA showed considerable variation among mothers and across time (Table 2). The concentration of both compounds was positively associated with age of the infants in our sample, but age was never a significant predictor of the concentration of either immune factor when included in the full model.

**Table 1.** Reported incidence of symptoms of illness experienced by 30 infants at 110 study visits, categorized by subtype

Infant symptoms	Incidence	Percent of total visits
Respiratory symptoms in past month	43	39.1
Gastrointestinal symptoms in past month	45	40.9
Any symptoms in past month	74	67.3
Current respiratory symptoms	47	42.7
Current gastrointestinal symptoms	12	10.9
Any current symptoms	53	48.2

**Table 2.** Descriptive statistics of milk compounds (*n* = 109 samples)

Compound	Mean concentration	Median concentration	SD concentration	Range of all samples	Range of individual maternal means
Lactoferrin (µg/mL)	2621	2378	1199	864–6581	2240–3948
sIgA (µg/mL)	648	556	379	183–2724	308–855

sIgA, secretory immunoglobulin A

Concentrations of lactoferrin and sIgA in milk were significantly related to the experience of symptoms of illness in infants (Tables 3 and 4). At higher concentrations of lactoferrin, infants were more likely to have experienced illness in the preceding month and more likely to experience illness in the subsequent month (odds ratios >1). This was true when all symptoms were analyzed together, as well as when gastrointestinal and respiratory symptoms were analyzed separately. At higher concentrations of sIgA, infants were less likely to have experienced illness in the preceding month and less likely to experience illness in the subsequent month (odds ratios <1). This was true for gastrointestinal and total symptoms, as well as respiratory symptoms in the preceding, but not subsequent, month. No relationships between the immune composition of milk and symptoms of illness concurrent with the study visit were significant.

**Table 3.** Relationships between milk immune compounds and symptoms of illness, categorized by month and symptom subtype: prior month symptoms of illness

Compound	Symptoms	Odds ratio	95% CI	*P*
Lactoferrin	Any	13.72	[2.71, 69.27]	0.0060
	Gastrointestinal	61.37	[12.27, 307.05]	0.0045
	Respiratory	9.46	[1.46, 61.44]	0.0342
sIgA	Any	0.03	[0.008, 0.135]	0.0045
	Gastrointestinal	0.02	[0.004, 0.120]	0.0342
	Respiratory	0.15	[0.032, 0.731]	0.0045

Results from GEE: relationships between milk immune compounds and symptoms of illness presented as odds ratios for the month preceding the study visit. The odds ratio represents the multiplicative change in odds of ‘success’ (illness) for a 1-unit increase in the explanatory variable (with everything else in the model held constant). In this case, a 1-unit increase in the explanatory variable is a 1-unit increase in the log-transformed values of the concentrations. All odds ratios associated with lactoferrin are >1, indicating a positive relationship with symptoms of illness in both time periods. All odds ratios associated with sIgA are <1, indicating a negative relationship with symptoms of illness in both time periods

ln, natural log. sIgA, secretory immunoglobulin A. CI, confidence interval.

All models include ln lactoferrin concentration, ln sIgA concentration, change in lactoferrin and sIgA concentrations from the previous month expressed as a percentage of previous month’s value, and infant sex. Outcome variable is binary, 0 = no symptoms experienced, 1 = symptoms experienced

**Table 4.** Relationships between milk immune compounds and symptoms of illness, categorized by month and symptom subtype: subsequent month symptoms of illness

Compound	Symptoms	Odds ratio	95% CI	*P*
Lactoferrin	Any	35.37	[1.65, 759.76]	0.0345
	Gastrointestinal	22.76	[3.75, 137.96]	0.0045
	Respiratory	15.12	[2.19, 104.48]	0.0135
sIgA	Any	0.04	[0.003, 0.515]	0.0293
	Gastrointestinal	0.04	[0.003, 0.478]	0.0293
	Respiratory	0.19	[0.029, 1.265]	NS

Results from GEE: relationships between milk immune compounds and symptoms of illness presented as odds ratios for the month following the study visit. The odds ratio represents the multiplicative change in odds of ‘success’ (illness) for a 1-unit increase in the explanatory variable (with everything else in the model held constant). In this case, a 1-unit increase in the explanatory variable is a 1-unit increase in the log-transformed values of the concentrations. All odds ratios associated with lactoferrin are >1, indicating a positive relationship with symptoms of illness in both time periods. All odds ratios associated with sIgA are <1, indicating a negative relationship with symptoms of illness in both time periods

ln, natural log. sIgA, secretory immunoglobulin A. CI, confidence interval.

All models include ln lactoferrin concentration, ln sIgA concentration, change in lactoferrin and sIgA concentrations from the previous month expressed as a percentage of previous month’s value, and infant sex. Outcome variable is binary, 0 = no symptoms experienced, 1 = symptoms experienced.

We calculated intraclass correlation (ICC) and 95% confidence interval for several variables to assess how much variance was attributable to individuals. The ICC [95% CI] for individual mothers’ lactoferrin and sIgA values (calculated using a generalized linear model with only maternal identity as a random factor) were 0.68 [0.51, 0.80] and 0.64 [0.48, 0.78], respectively. This indicates that a large portion of the variance in milk composition was attributable to individual mothers. Total monthly infant illness (calculated using a generalized linear mixed model approach with only infant identity as a random factor) had an ICC of 0.071 [0.002, 0.779], monthly respiratory illness had an ICC of 0.075 [0.002, 0.720] and monthly gastrointestinal illness an ICC of 0.229 [0.056, 0.598]. Less of the variation in infant illness month to month was attributable to individual factors, but these estimates are less precise.

## DISCUSSION

Consistent patterns emerged for the relationships between sIgA, lactoferrin and symptoms of illness. Almost all relationships between symptoms during the month prior to the interview and the month following the interview were significant. When significant, the odds ratios for sIgA were always <1 and the odds ratios for lactoferrin were always >1. Therefore, with everything else in the model held constant, as sIgA increases, it is associated with lower rates of illness. As lactoferrin increases, it is associated with higher rates of illness. These relationships indicate that there is some predictive power of milk immunofactors as biomarkers, predicting whether an infant is likely to be healthy or sick in the 2 months surrounding the sample. Relatively higher sIgA can be thought of as a biomarker for a healthy infant, and higher lactoferrin as a biomarker for a sick infant.

**Figure 1.**
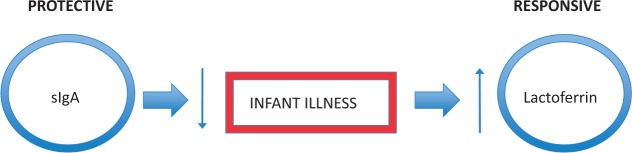
A schematic diagram of the Protective-Responsive Model of milk immune factors and infant illness. Milk is a complex bioactive fluid with the potential to protect against illness in the infant as well as to respond to illness with an adjustment of immune composition. This conceptual figure integrates the findings of the present study; sIgA follows the protective pattern, while lactoferrin exhibits a responsive role.

We find that the immune bioactives in breastmilk exhibit contrasting associations with infant illness when considered within the unified protective-responsive model (Fig. 1). The sIgA pattern illustrates the ‘protective’ paradigm, that higher levels of immune compounds in milk will protect the infant from illness. This is consistent with other studies that found a protective effect of sIgA against diarrheal illness [20, 21]. However, lactoferrin better fits the alternative ‘responsive’ pattern, that illness is associated with an increase in the production or transport of immune molecules to the mammary. While causality cannot be firmly established with the current data, these associations do lend support to the paradigms. These categorizations are not based on the temporal sequence of our own data *per se*, but from an understanding of the biology of the milk immunofactors and their function. When we see a positive association between a compound and illness, as we see with lactoferrin, we assume that illness has induced a rise in the compound, rather than the less intuitive interpretation that lactoferrin is causing symptoms of illness. Similarly, when we see a negative association between a compound and illness, as we do with sIgA, we infer a protective role, because the other interpretation (that infant illness suppresses maternal production of sIgA) makes less biological sense.

Some of our findings were unexpected. For instance, it is not immediately clear why symptoms of illness experienced at the time of the study visit were never significantly associated with milk sIgA or lactoferrin. It may be due to the small sample size (110 study visits from 30 infants), with a relatively small proportion of infants exhibiting symptoms of illness at any given time. There may also be a delay between the onset of symptoms and the adjustment of milk composition. In addition, the recent findings by the Hassiotou *et al.* [24]—that maternal or infant illness is associated with a rise in sIgA and no change in lactoferrin concentration—are seemingly in contrast to our own. However, in their study maternal infections were analyzed together with infant infections, and maternal infection was more strongly related to increases in milk leukocytes and humoral immunity compounds, including sIgA. Because of this and because of the different time scales of inquiry, it is not clear that our results are necessarily in opposition. In addition, as their study followed urban Australian women (who are assumed to have access to more hygienic conveniences), the different patterns that emerge may be related to the very different pathogenic environments experienced by our study populations.

We attempted to collect data about maternal experience of symptoms of illness, but women may not have been as forthcoming with their own symptoms as with those of their infants in the context of our face-to-face interviews (for instance, only one woman reported experiencing any gastrointestinal symptoms in 5 months). This is almost certainly due to underreporting, but regardless, did not introduce enough variability to test any relationships with milk composition. However, we expect maternal and infant exposure and concurrence of infection and symptoms to be strongly positively correlated. There are interesting theoretical implications raised by this assumption, which we were unable to test with the current data. If it is true that maternal and infant exposure to illness are positively correlated, it would be difficult to figure out *why* milk composition is altered; that is, to separate whether changes in milk immune composition were passively reflecting maternal circulating values, whether they were elevated to protect the mammaries from infection, or whether the adjustments were targeted directly toward supporting infant health. This would be a fascinating avenue for future research, but it is outside the scope of this article. Since the results presented here focus simply on the relationship between milk composition and infant illness, maternal illness should not affect the interpretation of our results.

The first evolutionary explanation that we investigated in an attempt to explain the opposite relationships that sIgA and lactoferrin exhibit with symptoms of illness is the idea that there may be a cost to the infant if lactoferrin is prophylactically elevated, perhaps related to its role in iron regulation. Whereas elevated sIgA may always provide a net benefit to the infant, elevated lactoferrin may result in a cost to the infant, such that mothers modulate lactoferrin secretion into milk to reflect current immunological needs of the infant. However, this explanation does not seem to be supported by the existing literature on this topic. Iron-saturated lactoferrin enhances the proliferation and differentiation of intestinal epithelial cells *in vitro* [29]. In addition, formula-fed infants who receive supplemental bovine lactoferrin were found to experience fewer lower respiratory tract infections and had higher hematocrits (indicating better iron status) at 9 months than those fed unsupplemented formula [23]. This study also reported a near-significant trend toward greater weight gain during the first 6 months of life for the lactoferrin-supplemented infants, similar to the findings of an earlier study in which lactoferrin supplementation was associated with significantly greater gains in height and weight in formula-fed infants [30]. Importantly, the infants in these studies were supplemented with bovine lactoferrin, and the infants were fed a dairy-based formula instead of human milk. Despite these caveats, it seems reasonably likely that higher lactoferrin is associated with many beneficial outcomes for the infant, weakening this particular argument as a potential explanation for the diverging patterns of sIgA and lactoferrin.

We cannot definitively explain the significance of the different patterns of sIgA and lactoferrin with the data from this study, but we offer two potential explanations to guide future work. One compelling explanation for the divergence is that there are different costs to the mother, the infant or both associated with the production and ingestion of these two compounds. For this to be the case, given our findings, we would expect that lactoferrin would be more costly to produce, or have more costly consequences associated with elevation, than does sIgA, and would therefore only be elevated when an infant experienced an illness and required additional lactoferrin to help fight the infection. Another possibility is that the lactoferrin and sIgA proteins have different functional lifespans, or control of production is regulated on different time spans. If lactoferrin is degraded or excreted more quickly, raising lactoferrin in an effort to prevent illness would be an ineffective or inefficient strategy, whereas responding to an existing infection with a rise in lactoferrin might be more effective. One piece of evidence to support the idea of differently timed regulation is that sIgA in milk is produced by maternal B-cells, which migrate to the mammary gland and release IgA into the secretory epithelium to be converted to sIgA and released into milk [17]. This process of migration and release is able to proceed more or less continuously, whereas lactoferrin has to be transcribed in mammary epithelial cells and may be more susceptible to the presence of pathogens, cytokines produced by other immune compounds, or transcription factors.

Our data have some limitations that prevent us from extending the protective-responsive model beyond its current logical framework into a more causal temporal understanding of the dynamics of milk immune composition. This is highlighted by the fact that the relationships between illness and milk composition that we uncovered are the same going forward and backward—i.e. milk immunofactors have similar associations with past and future illnesses. This would not be expected from a strictly temporal interpretation of these relationships and can probably be attributed to a number of factors relating to our methodology. The biggest limitation is the 1-month sampling regimen. Mothers were asked to recall whether their infants had experienced any of a number of symptoms in the previous month, or since the previous study visit. In addition to problems of self-report and recall, this led to symptoms experienced up to 4 weeks prior receiving the same statistical weight as more recent or ongoing symptoms. There is also the concern that certain infants may simply be sick more often than others, or may take longer to clear infections. A related concern is that certain mothers may produce consistently high or low concentrations of milk immunofactors (this appears to be true given the fairly high ICC values for sIgA and lactoferrin for individual mothers). Combined with the long sampling interval, these chronically ill infants or their chronically high-producing mothers may be obscuring the nuances of the temporal dynamics of the adjustment of milk composition with respect to an individual bout of illness. As an example, if an infant is ill both in the month preceding and the month following a given study visit, then it may be more likely that an immune factor will have a similar relationship to illness in both time periods than if the infant is only falls ill during one of the months. Similarly, many study visits may have fallen in the middle of a long bout of illness, which could inappropriately be classified as two separate bouts.

The protective–responsive model advanced in this article lends itself to the construction of explicit temporal predictions about the role and timing of lactoferrin and sIgA in symptoms of illness in infants. Future studies will benefit from a shorter sampling interval to allow for better resolution of the time scale on which the compounds and symptoms respond to each other, and researcher confirmation of the duration and severity of symptoms between infants would improve data consistency. Finally, the infants recruited to this study had a mean age of 244 days at the first study visit, all received supplemental foods, and nearly all received dairy products over the course of the study. Future studies should investigate the strength and direction of the relationships presented here among younger, exclusively breastfeeding infants.

### Conclusions and Implications

Our results add to the body of literature about the protective effects of sIgA against infant illness, particularly diarrheal illness. This study is also among the first to study natural variation of lactoferrin in human milk and its relationship to symptoms of infant illness. These findings are important as first steps toward a source of potential intervention against infant illness, particularly diarrhea, which is a leading cause of infant mortality worldwide. In addition to the public health implications of these findings, this study contributes to the dialogue in evolutionary biology surrounding breast milk composition. The data presented here demonstrate the importance of breast milk as a determinant of infant health, and suggest that mothers may be able to modulate milk composition to address the needs of their infants. The role of breast milk as a mediator between maternal and infant biology is worthy of further investigation. In particular, data that inform our understanding of the timeline of milk immune adjustment relative to infection and symptoms of illness, and about the relative efficacy of immune compounds in their protective and responsive roles would be useful in expanding the conceptual model presented in this article into a comprehensive description of milk’s dynamic role in infant immunity.
